# Identifying factors in the provision of intravenous stroke thrombolysis in Malaysia: a multiple case study from the healthcare providers’ perspective

**DOI:** 10.1186/s12913-023-10397-8

**Published:** 2024-01-05

**Authors:** Wen Yea Hwong, Sock Wen Ng, Seng Fah Tong, Norazida Ab Rahman, Wan Chung Law, Sing Keat Wong, Santhi Datuk Puvanarajah, Aisyah Mohd Norzi, Fiona Suling Lian, Sheamini Sivasampu

**Affiliations:** 1 Institute for Clinical Research, National Institutes of Health, Ministry of Health Malaysia, Shah Alam, Selangor, Malaysia; 2 Julius Center for Health Sciences and Primary Care, University Medical Center Utrecht, Utrecht University, Utrecht, the Netherlands; 3 Department of Family Medicine, Universiti Kebangsaan Malaysia, Kuala Lumpur, Malaysia; 4 Neurology Unit, Department of Medicine, Sarawak General Hospital, Ministry of Health Malaysia, Kuching, Sarawak Malaysia; 5 Department of Neurology, Hospital Kuala Lumpur, Ministry of Health Malaysia, Kuala Lumpur, Malaysia; 6 Clinical Research Centre, Penang General Hospital, Ministry of Health Malaysia, Penang, Malaysia

**Keywords:** Acute stroke care, Intravenous thrombolysis, Developing countries, Translational research, Determinants, Factors

## Abstract

**Background:**

Translation into clinical practice for use of intravenous thrombolysis (IVT) for the management of ischemic stroke remains a challenge especially across low- and middle-income countries, with regional inconsistencies in its rate. This study aimed at identifying factors that influenced the provision of IVT and the variation in its rates in Malaysia.

**Methods:**

A multiple case study underpinning the Tailored Implementation for Chronic Diseases framework was carried out in three public hospitals with differing rates of IVT using a multiple method design. Twenty-five in-depth interviews and 12 focus groups discussions were conducted among 89 healthcare providers, along with a survey on hospital resources and a medical records review to identify reasons for not receiving IVT. Qualitative data were analysed using reflective thematic method, before triangulated with quantitative findings.

**Results:**

Of five factors identified, three factors that distinctively influenced the variation of IVT across the hospitals were: 1) leadership through quality stroke champions, 2) team cohesiveness which entailed team dynamics and its degree of alignment and, 3) facilitative work process which included workflow simplification and familiarity with IVT. Two other factors that were consistently identified as barriers in these hospitals included patient factors which largely encompassed delayed presentation, and resource constraints. About 50.0 – 67.6% of ischemic stroke patients missed the opportunity to receive IVT due to delayed presentation.

**Conclusions:**

In addition to the global effort to explore sustainable measures to improve patients’ emergency response for stroke, attempts to improve the provision of IVT for stroke care should also consider the inclusion of interventions targeting on health systems perspectives such as promoting quality leadership, team cohesiveness and workflow optimisation.

**Supplementary Information:**

The online version contains supplementary material available at 10.1186/s12913-023-10397-8.

## Background

The introduction of intravenous thrombolysis (IVT) using recombinant tissue plasminogen activator (r-TPA) has created a paradigm shift in the management of ischemic stroke. A decrease in the odds of disability at 3–6 months was proven following IVT [1]. Similarly, its benefits persisted in reducing the risk of mortality at long term [2].

Translation of this evidence-based treatment into clinical practice however, remains a challenge. The rate of IVT varied where high-income countries were shown to have a higher uptake at 20.6% in the Netherlands, 15.0% in Sweden and 11.9% in the United Kingdom [3] as compared to an average of 3% across low and middle-income countries (LMICs) [4]. Unexpectedly within a country, large regional differences was also reported; local IVT rate varied between 6 and 35% in the state of Hesse in Germany [5].

Overall, reasons for the low rates have been systematically studied and they ranged widely from patients’ delayed presentation to the hospital to in-hospital factors including healthcare providers’ familiarity and competence in IVT, limited infrastructure and human resources, complex guidelines, and poor organisational support [6, 7]. As majority of these studies were conducted in high-income settings, it is imperative to understand the reasons for poorer rates of IVT in LMICs. This is especially when 89% of deaths and disabilities from stroke occur in this region [8]. These reasons are expected to differ given a vast contrast from the provision of healthcare systems, drug subsidies and reimbursements to socioeconomic statuses of the residents.

Malaysia is one of the LMICs with a two-tiered healthcare system, consisting of a heavily subsidised public facilities and a mainly out-of-pocket private healthcare. The use of r-TPA for IVT in stroke has been approved by the local regulatory since 2012, with recommendations being made available in the local clinical practice guidelines [9]. With existing universal health coverage in Ministry of Health (MOH) hospitals, treatment provided to Malaysian residents is at minimal charges. Nevertheless, it was recognised that implementation of this service nationwide was not easy. The average IVT rate among ischemic stroke patients in MOH hospitals was low at 1.6% in 2018 (Hiew FL: Stroke Thrombolysis Survey in Ministry of Health Malaysia in 2018, unpublished data) but interestingly, the rates differed widely across different hospitals. Understanding similarities and differences in the challenges faced by respective hospitals and how these challenges were managed is crucial to plan for effective and targeted strategies.

Therefore, this study aimed at identifying factors that influenced the provision of IVT and the variation in its rates across public MOH hospitals in Malaysia, from the perspective of healthcare providers. The identified factors would be examined within the hospitals and cross-compared against the basis of an implementation science framework to identify their influence in the adoption of IVT into clinical practice.

## Methods

### Research team

The main research team members comprised of SFT, a male professor in family medicine with vast experience in qualitative research and four female researchers: WYH, SWN, NAR, and AMN. Under the guidance of SFT, they were trained in qualitative research and were responsible for the conduct of both the qualitative and quantitative data collection methods. WYH and NAR also have higher qualifications in epidemiology with experiences in stroke-related research.

None of the researchers had personal relationships with the participants. Several participants have worked with the researchers for other non-related studies. The researchers are not directly involved in the care of stroke patients and therefore, have impartial views on the discussed topic. Participants were recruited after they consensually agreed that the research was conducted with the purpose to seek ways to improve the provision of IVT in Malaysia.

Other members of the team are neurologists with experience in stroke care (SDP, WCL, SKW) and SS, a public health specialist and health systems researcher. They were involved in study design and providing clinical input in analysis and interpretation of data.

### Study design

We used a multiple case study design to allow an in-depth exploration on a phenomenon in its natural setting. As a continuity to our published article depicting the success of IVT from a single hospital [10], a multiple case design approach has allowed a more comprehensive exploration of the research question by considering the similarities and differences across case studies following detailed understanding of each case.

This study is underpinned by an implementation science framework called the Tailored Implementation for Chronic Disease (TICD), which guides understanding on the determinants of implementation change in clinical practice and is consolidated from 12 reviews of implementation determinants [11]. Apart from the relevance of its application as a screening tool to identify determinants of implementation change, this framework has also been used in a number of similar studies which focused on the provision of acute stroke care [6, 12]. The determinants of practice are categorised in seven domains which include factors related to individual health professionals, professional interactions, guidelines, incentives and resources, patients, capacity for organizational change, and social, political and legal [13]. The TICD was applied as a guide to develop the interview guides and to conduct the initial coding for data analysis.

### Case definition

Hospitals were purposively sampled based on the rate of IVT ranked from highest to lowest among the cases, which refers to all MOH hospitals with neurologists at study initiation (*n* = 13) in 2019. Considering that policies to disburse allocation of financial and human resources should be similar across MOH hospitals, three hospitals with differing rates of IVT (highest provision, average, and least to no provision) were selected. The hospital with the highest rate of IVT was referred to as “Hospital Z,” one with an average rate was named “Hospital Y” whereas the hospital of which IVT was not offered was called “Hospital X”. Overall, the selection of these three hospitals with different IVT rates was deemed to be able to represent a spectrum of challenges and experiences which would have led to the variations in the rates.

### Participant selection

Our inclusion criteria were healthcare providers who were directly involved in IVT and senior administrators who were authorized to make decisions on the provision of IVT in respective hospitals. The provision of IVT involves healthcare providers from different professions including mainly: 1) neurologists; 2) physicians or medical officers from emergency department (ED); 3) physicians or medical officers from medical department; 4) radiologists or radiographers; 5) nurses from ED; 6) medical assistants (MA) from ED; 7) nurses from neurology department; 8) pharmacists; and 9) head of medical department and/or hospital director. Medical assistants are typically tasked to care for patients with limited supervision and in Malaysia, patient triaging is one of their main tasks [14].

An average of 9–17 in-depth interviews (IDIs) or 4–8 focus group discussions (FGDs) are needed to reach saturation [15]. To ensure saturation and at the same time, allowing sufficient variations of working experiences based on their respective roles, we included at least one healthcare provider from each profession for the interviews and at least two for the focus group discussions. We also verified that those included had at least 6-months experience in the study site to allow their sharing of experience to reflect fairly on the true situation on the ground.

Participants were recruited through contacts with relevant department heads in each hospital. From their recommendations, potential participants were purposively selected. Snowballing of more participants were carried out following suggestions from the participants. Written informed consent were obtained from each participant prior to the IDIs or FGDs. We verbally asked for a reconfirmation of the consent at the start of the interviews or FGDs. In total, 95 participants were invited and three declined for undisclosed reasons. Another three could not attend the interview because of hospital admission (*n* = 1) and emergency calls (*n* = 2).

### Data collection

In our study, multiple sources for data were used as a strategy for triangulation:

#### In-depth interviews (IDIs) and focus group discussions (FGDs)

The main data collection strategies were IDIs and FGDs which were conducted in a semi-structured manner. A total of 25 IDIs and 12 FGDs were conducted (Table 1). In-depth interviews were conducted for the professionals where their personal experiences and perceptions towards IVT were comprehensively explored. Focus group discussions were conducted for the supporting providers which included the nurses, MAs, radiographers, and pharmacists, to spur discussions via peer interactions on the experiences and challenges in supporting the professionals for the conduct of IVT among participants with common experiences. Perspectives from FGDs were expected to provide a greater understanding to the programme implementation contextually. Furthermore, moderators in FGD were also more likely to be perceived as less authoritative than IDI interviewers and therefore, would lessen the fear amongst the supporting providers to speak especially on potentially less favourable experiences. English was the main language used, except for the FGDs where both English and Malay languages were used for easier understanding. The number of participants in the FGDs ranged from 2 to 15 in a group. Each IDI and FGD lasted about 45 to 60 min with no repeats.

**Table 1:** Distribution of participants by profession and types of interviews conducted

Participants	Number of participants			Types of interviews
	**Hospital Z**	**Hospital Y**	**Hospital X**	
Neurologists	2	3	1	In-depth interview
Hospital Director/head of medical department	2	1	1	In-depth interview
Physicians/medical officers from emergency department	2	4	2	In-depth interview
Physicians/medical officers from medical department	2	0	0	In-depth interview
Radiologists/radiographers	5	2	2	In-depth interview/ Focus group discussion
Nurses from ED	4	15	3	Focus group discussion
Medical assistants (MA) from ED	4	8	3	Focus group discussion
Nurses from neurology department	6	10	0	Focus group discussion
Pharmacists	2	3	2	Focus group discussion
**Total**	**29**	**46**	**14**	

Interview guides for both IDIs and FGDs were developed using the basis of the determinants from the TICD framework. Selection of relevant domains and determinants to the provision of IVT were made before applying them to develop the guides (Additional files 1 and 2). The guides were then adapted for different profession to suit their scope of work as well as opinions from qualitative experts. The guides were tested among a sample of healthcare providers who were not from our study sites and were adapted and finalised following their feedback.

This study was initiated just before the Covid-19 pandemic struck in early 2020 and thus, data collection was impacted by national lockdowns and subsequent restrictions of national standard operating procedures. Apart from a delay in data collection, strategies to collect data were also improvised. In-depth interviews and FGDs were conducted physically in confidential settings within one hospital prior to the first Covid-19 lockdown. Data collection for the other two hospitals had to be shifted to a subscribed video conferencing platform.

Both IDIs and FGDs were recorded using both audio and video recordings with field notes taken to catch non-verbal cues and important responses. All records were transcribed verbatim according to the language used in the interviews and FGDs and checked randomly for accuracy against the audio recording. We did not return the transcripts to participants for comments.

#### Surveys and medical records review

To augment rigor of findings from the qualitative data, a survey and a medical records review were carried out. The survey focused on quantifying available hospital resources by relevant departments in respective hospitals. Information collected included total number of beds, total number of ischemic stroke patients, stroke services, access to diagnostic imaging and details on the provision of IVT. The medical records review was conducted to explore the reasons for not receiving IVT among ischemic stroke patients, specifically to examine the contribution of patient-related factors. From each hospital, a systematic sampling of every fifth patient from the list of ischemic stroke patients admitted from June to December 2019 was conducted. Medical or stroke registry records were then reviewed to identify reasons why patients did not receive IVT. In total, 105 patient records were included in Hospital Z, 94 from Hospital Y and 106 from Hospital X.

### Data analysis

NVivo 12 software was used for compilation and management of the transcripts [16]. WYH and SWN conducted the initial line-by-line coding independently by deductively applying the 57 determinants from the TICD framework as a guide to identify potential factors affecting a programme implementation. Nevertheless, the coding was neither restricted to the available determinants nor its specific labelling or categorisation from the published framework. The codes were subsequently compared, coded, and classified into relevant domains by inductively deriving them from the context of stroke care in a low- and middle-income setting to ensure an in-depth analysis of the texts rather than solely labelling them with the available TICD determinants. Specifically, determinants which influenced the variations observed across the participating hospitals were also accounted for during the coding and categorisation. The relationship between these domains were also derived in the similar manner. Besides, codes were grouped by the participants’ profession for a clearer understanding of the information provided in its context. The coding exercise was done separately for each hospital, although newly derived codes were shared for similar findings derived across the hospitals.

Similarly, findings from the surveys and medical record reviews were analysed separately for each hospital and presented in descriptive statistics.

The main analysis involved triangulating the qualitative and quantitative data within each participating hospital before considering their similarities and differences via a cross-case comparison. Results from the quantitative data were used to support and converge the evidence on the extent of the contribution of certain domains; medical records review to strengthen findings on mainly patient-related factors whereas the survey to verify findings in terms of availability of resources. Findings were subsequently corroborated with the variation of the IVT rates before iteratively adapted with the purpose to develop the theory on factors influencing the provision of IVT. This process involved several in-depth discussions among the main research team members on explanation of the qualitative findings, whether the quantitative results were consistent with the main findings as well as the choice of quotations to be presented for each case and across cases. Quotations in Malay language was translated into English for reporting purposes. Finally, we verified the credibility of our findings by conducting rounds of feedback with content experts and peers who were not directly involved in data analysis.

## Results

### Characteristics of stroke care services

Table 2 shows a summary of the characteristics of stroke care services for the cases studied. On average, rates of IVT in Hospital Z increased, with a range between 4.8% in 2014 and 20.8% in 2018. A dedicated stroke team is available, although the neurologists are remotely consulted after office hours. Patients who present with acute neurological deficit in ED will be triaged using a fast-tracked standardised IVT workflow before being sent for CT imaging in a different building. Eligible patients will be sent to the acute stroke unit for thrombolysis.

**Table 2:** Summary of the characteristics of stroke care services by each participating hospital^a^

	**Hospital Z**	**Hospital Y**	**Hospital X**
**Hospital admission**			
Total number of beds in hospital	1057	2125	1163
Availability of neurology ward	No	Yes	Yes
Number of ischemic stroke patients were admitted to the hospital between 2013 and 2019	116—610	461—1148	530—784
**Stroke services at emergency department**			
Protocol for rapid triage of patients presenting with acute stroke in emergency department	Yes	Yes	No
Prioritisation of CT scan for suspected stroke patients	Yes	Yes	No
**Specialised stroke care**			
Management of stroke cases to be led by	Neurology unit under the Department of Internal Medicine	Department of Neurology	Department of Neurology
Number of neurologists	2	7	2
Availability of neurology trainees	No	Yes	Yes
Dedicated acute stroke unit	6-bedded cubicle within medical ward	No	No
Dedicated stroke team	Yes	No	No
**Access to diagnostic imaging/lab**			
Availability of CT machine in ED	No	No	No
Plain CT brain	Yes	Yes	Yes
Accessibility for CT brain	24 h	24 h	24 h
CT angiography	Yes	Yes	Yes
Accessibility for CT angiography	24 h	24 h	24 h
MRI services	Yes	Yes	Yes
Accessibility for MRI	Monday to Friday, 8am—5 pm	extended hours including weekends	Monday to Friday, 8am—5 pm
**Pharmacotherapy/management**			
Availability of IVT for ischemic stroke patients	Yes	Yes	No
Year of starting IVT	2013	2013	na
Year of starting 24 h access to IVT	2015	2019	na
Rate of IVT			
2013	5.2	2.4	na
2014	4.8	2.8	na
2015	11.1	1.8	na
2016	14.1	2.2	na
2017	15.7	1.5	na
2018	20.8	2.8	na
2019	15.1	4.9	na
Location of provision of IVT	Acute stroke unit	CT suite and/or Emergency Department	na
Mechanical thrombectomy	No	Yes	No

*CT* Computed tomography, *IVT* Intravenous thrombolysis, *NA* Not available

^a^data were collected between year 2020 to 2021; therefore access and availability may differ to current situation

As opposed to Hospital Z, Hospital Y has a separate Department of Neurology with a neurology ward. The rate of IVT were rather constant at 1.5 – 2.8% before it rose to 4.9% in 2019. Instead of a dedicated stroke team, the neurologists on call would attend to stroke activation calls. Intravenous thrombolysis will be provided either at the CT suite or ED due to the lack of an acute stroke unit at the point of interview.

Intravenous thrombolysis was not offered in Hospital X and therefore, there is no protocol for stroke activations or prioritisation for CT scans for suspected ischemic stroke patients.

### Determinants of the provision of IVT

**Fig. 1:**
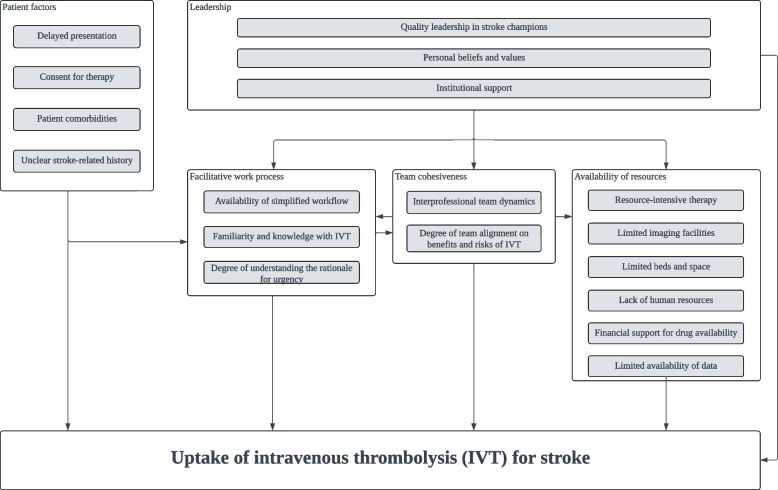
Relationship between contributing factors and its respective overarching domain to the provision of intravenous thrombolysis. Grey boxes refer to factors within the specified domain

This cross-case analysis identified 5 overarching domains adapted from the TICD framework, which attributed to the provision of IVT in this country. In our single case study, cohesiveness among team members was particularly striking within that hospital, resulting in leadership being embedded as one of the contributing factors to team cohesiveness [10]. Nevertheless in this cross case comparison, the pivotal role of leadership emerged as one of the key factors to be independently considered. Figure 1 explains the relationship between the domains.

#### Leadership

*Quality leadership in stroke champions*

Participants in Hospital Z shared that having a leader that is passionate and committed in championing stroke care was the key facilitator in delivering acute stroke care services: *“I think the person doing it is very important, Dr A. I can see his dedication. I think we can all share his enthusiasm* (Z-11; IDI)*”*. Furthermore, the neurologists were praised for being hands-on and were willing to be consulted directly which allowed other healthcare providers to develop confidence in handling stroke cases. Motivation to continue providing IVT has also been attributed to how the neurologists often credited the ground staff for achievements earned from providing IVT: *“Dr A does share with us some awards that they achieved. (This brings) some positive reinforcements for the radiographers. They are the ones who do 24 h shift to scan the patients* (Z-11; IDI).”

In Hospital Y and X, a lack of passionate stroke champions was consistently brought up: *“Our key challenge over the years has been that we have had many neurologists trained in stroke but they have all left for greener pastures* (Y-05; IDI)*”* and *“No matter how hard we push for thrombolysis, but let’s say the (primary) team don’t agree with this, we can’t do anything* (X-02; IDI).*”*

*Personal beliefs and values*

Enthusiasm and motivation among the leaders in Hospital Z were evident: *“I always believe I wanted to do for others what I wanted them to do for me. I think that is the main drive* (Z-01; IDI)*”* and *“With the introduction of treatments, you can help patient(s) to live (an) independent life. That is a good motivation for me personally because you know that your work makes a difference* (Z-07; IDI)*.”*

In Hospital Y and X however, there were contradicting intentions to provide IVT: *“There are two groups of doctors. One will find every single reason to thrombolyse the patients. Another will find every single reason not to thrombolyse patients* (Y-10; IDI).*”* Importantly, the narrow therapeutic window exerted pressure on the leaders from Hospital Y: *“The oldest school are more conservative. Neurology even though it is a tough field, there was an opportunity for you to go back home in the night. Stroke changes it the way it changes for cardiologist* (Y-05; IDI)*”.*

In Hospital X, there was an inclination to manage stroke patients using an interesting but rather contrasting approach: *“We found that by using this (functional medicine) approach, a lot of patients usually recover faster* (X-01; IDI)*.”* Use of thrombolysis was therefore, seen as not the only strategy to provide optimal stroke care: *“Irrespective of what method we use, as long as patient gets better, it is a good approach because the ultimate aim (is for the patients) to improve* (X-01; IDI)*.”*

*Institutional support*

Support from higher authorities and other departments was recognised as an important factor in service establishment and delivery. Participants from Hospital Z shared positive experiences on this where the higher authorities were appreciative of their effort to provide IVT and had innovatively maximised the existing human resources and facilities to support the service, despite having limitations: *“There are times that resource is an issue. Then, we (would) look at how to juggle the resources to ensure the best outcome* (Z-13; IDI)*”.* Contrastingly, there were mixed reactions from those in Hospital Y and X. Some refused to comment but there were also good reflections received: *“I think in all levels, from the Director to my head of department (HOD), the neuro HOD, everybody is working together with the same aim. Basically, we all try to increase the number of patients that we thrombolyse* (Y-02; IDI)*”.*

#### Team cohesiveness

*Interprofessional team dynamics*

Effective engagement among team members

Providing opportunities for communication through interdisciplinary meetings and building rapport were observed to lead to effective team dynamics in Hospital Z. *“I spent a lot of time going down to the radiology department, talking to the radiologist and making myself known to them* (Z-07; IDI)*”* and *“Communication with each other, interdisciplinary meetings, and discussions. All that to settle problems* (Z-12; IDI)*.”*

Effective engagement was also made possible with having approachable leaders: *“Everyone has been approachable. That certainly helped in getting and pulling everyone to work together as a team* (Z-07; IDI)*.”* Besides, having effective engagement has also led to the expansion of IVT to nearby district hospitals without neurosurgeons. The neurosurgeons in Hospital Z were willing to take in patients from these hospitals should complications from IVT arose.

In Hospital Y, several participants concurred to the importance of effective engagement to resolve issues that may potentially prevent the provision of IVT within a department. One doctor illustrated (Y-02, IDI): *“We have an open dialogue. Sometimes if they encounter a problem in my department, they just text me straight and I address it. It does not get delayed or dragged on.”* Gaps within dynamics of the team however, mainly stemmed from poor inter-department communication. Similarly, difficulties to initiate IVT in Hospital X was also attributed to the lack of communication across departments (X-02; IDI): *“It is mainly due to the lack of communication between departments. If one party cannot agree with another party’s opinion, then nothing can be done.”*

Joint ownership of responsibility

Trust was a fundamental aspect in nurturing joint ownership of responsibilities in Hospital Z. To cope with the lack of human resources, the neurologists trusted, empowered, and privileged other healthcare providers to independently handle certain tasks within the IVT process: *“We do not have trainees. So, a lot of our bout (are) being covered by (medical) physicians (and) medical officers* (Z-07; IDI)*.”* Likewise, the radiologists trusted the neurologists to interpret CT images for stroke patients: *“We are okay with them (neurologists) interpreting the scan. For them, (they need) to have immediate (interpretation), because (they) need to act on the scan finding(s)* (Z-11; IDI)*.”*

Besides trust, being interdependent was demonstrated through sharing of the task to send patients to CT suite. One MA explained (Z-06–01; FGD): *“Upon stroke activation, sometimes the stroke team or the ED housemen will push the patients for CT scan. They do not rely on MAs, nurses, or porters.”* Furthermore, a positive attitude towards the workload from IVT was observed: *“We only claim off hours. We do not claim (money)* (Z-02–02; FGD)*.”*

Encouragingly, this working culture was also observed in Hospital Y and X, especially among the ED physicians: *“We have been giving thrombolysis for MI patients, just that (this requires) more close monitoring and clear-cut indications. If (if) you can establish (the service) at least half-way, we can try to facilitate* (X-02; IDI)*.”*

Despite such positivity, mixed opinions about IVT remained across departments in all three hospitals. Several participants expressed hesitancies: *“Most of us are not there yet. To say that we would take over the decision to thrombolyse, not all of us are used to it* (Z-04; IDI)*.”* Some were concerned about the extra workload the service would entail (X-05; IDI): *“I can foresee if the stroke services (start), there’s a (need) for an on-call radiologist. They will need to work harder.”* Others were not comfortable with having healthcare providers from other professions to share their roles. One doctor noted (Y-14; IDI): *“I think eventually thrombolysis is going to be like giving streptokinase (to MI patients). But you must understand the heart and the brain are two different things. If a general physician wants to do it, they still must consult a neurologist.”*

Qualities of effective feedback

Despite not having regular feedback on patient outcomes or work-related performances, Hospital Z participants agreed that there were avenues to provide and receive feedback if the need arises. Negative feedbacks were surprisingly accepted as constructive encouragements to motivate the ground staff to do better: *“They (the neurologists) get upset when it (stroke) is missed but not overly upset. Appropriately upset. So, we try not to miss. The culture is such that we want to do it well* (Z-09; IDI)*”* and *“Because we know every (scolding) is for the sake of the patients. (In) the end it is just to make the patients better. It is nothing personal* (Z-11; IDI)*.”* Importantly, the participants shared how information from feedbacks would be transferred to them although the initial discussion involved only the higher authorities. Contrastingly, although regular audits on data for stroke patients were available in Hospital Y, a concern of not receiving feedback was raised: *“(For) multi-disciplinary (meetings), the bosses are the ones attending. But information (from the meetings) does not reach the ground staff although it is the ground staff who are attending to these stroke cases* (Y-13; IDI)*.”*

*Degree of team alignment on benefits and risks of IVT*

Despite having different professions, majority of the ground staff and professionals in Hospital Z shared aligned values on the benefits of IVT on patients’ outcome: *“We have seen quite a lot of patients that come in very bad then (could) go home walking. Their quality of life is very much improved* (Z-14; IDI)*.”* Echoed by a nurse (Z-02–03; FGD): *“There are some patients with power zero who can improve to three. That makes us satisfied.”* Parallel to that was their perspective on the risk of bleeding after IVT: *“We are talking about less than 3% (of symptomatic haemorrhagic transformation that potentially might require intervention). I think the benefit outweighs the risk* (Z-01; IDI)*.”*

The scenario appeared different in Hospital Y and X where conflicting arguments on the benefits and risks from IVT arose. While many believed that it would lead to beneficial outcomes, a few remained less comfortable with the rapid expansion of leniency in the recommendations provided. Stressing the importance of careful patient selection, one doctor mentioned (Y-14; IDI): *“Some of these patients theoretically may have done well even if you do not thrombolyse. I have enough patients that I do not thrombolyse. But they still do okay. And there are some patients who were thrombolysed, we wished in retrospect we had not thrombolysed.”* This sentiment was shared in Hospital X: *“Certain groups that they thrombolysed, the scan is within normal limits. How sure are we that this is not TIA because by definition, TIA spontaneously recovers within 24 h* (X-01; IDI)*?”.*

#### Facilitative work process

As IVT was not offered, this factor was not analysed in Hospital X because IVT workflow naturally did not exist and participants’ opinions on knowledge, skills and degree of urgency would be hypothetical rather than based on previous experiences.

*Availability of simplified workflow*

One of the main issues for identification of stroke patients were due to their atypical symptoms or concurrent presenting complaints. Consequently, some of these patients would be mistriaged and this caused either a delay or inability to provide IVT. In response to this, Hospital Z established a lenient and simplified workflow to activate thrombolysis: *“We changed the (triaging) protocol to acute neurology deficit. We are very lenient for them (ED) to activate and call us* (Z-01; IDI)*.”* Despite having more false alarms initially, the percentage of patients who received IVT after a stroke activation increased from 53.9% in 2016 to 79.7% in 2018, indicating an improvement in terms of identification of stroke patient at triage with time [10].

The usual workflow for referrals from district hospitals without CT machine to send patients to Hospital Z was also simplified: *“For district hospital without scan, we encourage them that if they think it’s a stroke, call us and bypass (their own hospital) to come (straight)* (Z-01; IDI)*.”* Echoed by a doctor from Hospital Y: *“If it is an ambulance call, the team goes to the site and assess the patient. If they establish an acute stroke, they will inform the control centre who would inform the ED team to standby with pre-filled forms and activate stroke calls* (Y-02; IDI)*.”*

In both hospitals, efforts to facilitate the IVT workflow were observed. These include having standardised documents such as acute stroke protocols, stamps for urgent CT requests, and templates for ED clerking and reporting CT imaging. Use of technology has also been applied to aid sending and interpreting CT images.

**Fig. 2:**
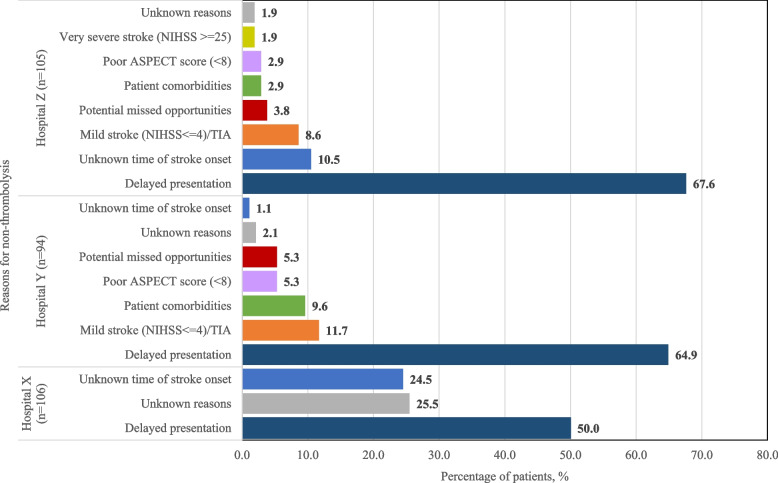
Reasons for non-thrombolysis among ischemic stroke patients in respective participating hospital. TIA: Transient ischemic attack; NIHSS: National Institutes of Health Stroke Scale; potential missed opportunity includes those with delayed referral to CT imaging and delayed assessment by stroke team. Unknown reasons in Hospital X refers to potentially eligible patients by timing but may have other reasons to not receive thrombolysis

Figure 2 shows 3.8% of patients who missed the opportunity to receive IVT in Hospital Z. Medical records review revealed potential in-hospital workflow issues where delays in referral for CT imaging and assessment by stroke team occurred, with one being an inpatient stroke. The percentage was higher in Hospital Y at 5.3%, with similar in-hospital workflow-related concerns.

*Familiarity and knowledge with IVT*

Being familiar with respective roles and having hands-on experiences through non-structured training such as tagging a senior doctor have also led to competent and knowledgeable staff. One doctor explained (Z-04; IDI): *“Over time, we get comfortable with it that it becomes a reflex. You say that this is stroke, people would know what to do.”* A radiographer agreed (Z-08–03; FGD)*: “It was my seniors who taught me. They also explained the importance of IVT.”*

A different situation was observed in Hospital Y. The ground staff admitted that difficulties to identify stroke patients occurred with a lack of knowledge and experience. Emphasized by a medical assistant (Y-03–05; FGD): *“Sometimes the triager overlooks and would triage stroke patients as having hypoglycemia, ignoring the need to assess BEFAST. The patients will be sent to yellow zone.”*

*Degree of understanding the rationale for urgency*

It was commonly agreed in Hospital Z that patients for IVT must be treated urgently: *“If they activate thrombolysis, we drop everything and attend. We do not hesitate to attend* (Z-09; IDI)” and *“No matter how busy we are, we will try to expedite the process to send patients (for) CT and activate the stroke team* (Z-10; IDI)*.”*

Contrastingly, participants from Hospital Y shared how different their priorities were despite similarities in the urgency for time-dependent therapies between cases of acute stroke and myocardial infarction: *“If a patient (has) myocardial infarction, all the staff will have adrenaline rush to attend the patient. For acute stroke however, we still do not have the adrenaline rush (*Y-06; IDI*).”* The lack of urgency was similarly noted in the radiology department (Y-13; IDI): “*They (the radiographers) thought they should finish up the regular appointments first before slotting in stroke patients. Even some doctors were not aware. They thought it is okay to wait.”*

#### Patient factors

One factor which was consistently identified as a barrier to provide IVT across all three hospitals was patient-related factors; the largest attributable factor being delayed presentation.

*Delayed presentation*

Hospital Z recorded 67.6% of ischemic stroke patients who did not receive IVT arrived at the hospital outside the narrow therapeutic window time, followed by 64.9% in Hospital Y and 50.0% in Hospital X (Fig. 2). Among the suggested reasons for delayed presentation include low public awareness of stroke symptoms recognition and the availability of a time-dependent therapy, logistics, and patients’ preferences on where to get treatment.

As distance from district hospitals without CT machines to Hospital Z is far, one pharmacist explained (Z-03–01; FGD): *“Our patients in (this region) (has) transport(ation) issues and tend to present very late.”* Concurred by another (Y 08–03; FGD): *“A lot of people do not even know (that) if they presented early, they can be given thrombolytic therapy.”* In addition, the culture of seeking treatment from traditional healers remained prominent in the country: *“They (patients) were in denial that they had a stroke. They asked for AOR (discharge at own risk) and go for massages and traditional treatment (*X-04–01; FGD*).”*

*Consent for therapy*

Although few patients would refuse IVT, the participants explained how delays may occur during consent taking due to language barriers or when indecisive patients requested for discussions with family members. Doctors from Hospital Z and Y shared their experiences on the importance of simple explanations to patients and their family members: *“If you tell the family members that we give (thrombolysis) there is a chance you will improve and, if we do not, most likely you will remain like this, most of the time, they are quite receptive even though you tell them there is a risk of bleeding* (Z-14; IDI).”

*Patient comorbidities*

Figure 2 shows that patient comorbidities attributed to the reasons for excluding eligible patients from IVT. In Hospital Z, 2.9% of the patients who did not receive IVT had uncontrolled blood pressure levels or seizure upon onset of stroke. Aside from blood pressure control, patients’ medical history of recent stroke or risk of bleeding constituted 9.6% of the reasons for non-thrombolysis in Hospital Y. Besides that, delays could also be caused by patients’ poor condition upon arrival, potentially excluding them from the opportunity to be thrombolysed.

*Unclear stroke-related history*

Unclear patient history and language barrier could complicate the process of identifying eligible ischemic stroke patients for IVT and subsequently delay referrals. One MA said (Z-06–04; FGD): *“Sometimes the family members could not give an exact time and do not know when symptoms have started.”* This was agreed by a doctor (Y-07; IDI): *“Language would be an issue especially for older patients. A lot of times we cannot really get the exact time of onset and their pre-morbid.”* Likewise, this finding was evident from the medical records where patients with unknown onset constituted 24.5% of ischemic stroke patients in Hospital X, 10.5% in Hospital Z, and 1.1% in Hospital Y (Fig. 2).

#### Availability of resources

*Resource-intensive therapy*

Stroke IVT is acknowledged to be resource intensive. One doctor explained (Z-01; IDI): *“Thrombolysis is labour intensive and we must respond very quickly. It is every 15 min monitoring. It is costly. We need CT scan. We used to have to call radiologist(s) to get the permission, then we get patient consent for CT and then push their way to CT scan room which is always not next to ED. And then have to interpret CT. There is always a reservation among neurologists in Malaysia, because it is labour intensive.”*

*Limited imaging facilities*

Participants from all hospitals concurred on the lack of CT machines to accommodate imaging requests in their respective hospital: *“Because (there) is only one CT scan, you are competing against poly-trauma cases. So, it sometimes could be a delay before they call you* (Y-05; IDI)*.”* A lack of CT imaging facilities in ED was also brought up as a concern because patients who require CT imaging would have to be transferred to CT suite, attributing to valuable time lost:* “Patients who needs CT scan must be sent all the way up to the first floor. The lift is not a dedicated lift for patients* (Y-05; IDI)*.”* In Hospital Z, challenges were also mentioned with the limited availability of MRI slots for extension of window therapy for wake-up stroke: *“There is only one MRI machine and the queue is extremely long for MRI (even) for normal standard appointments* (Z-07; IDI)*.”*

*Limited beds and space*

Issues regarding limited beds and space was expressed by most participants. Neither conducting IVT nor accommodating an imaging machine in ED of Hospital Z was possible due to restricted space. Nevertheless, the neurologists have taken the initiative to establish an acute stroke unit comprising of a cubicle in their medical ward and purchased a weighing bed which is reserved for patients undergoing IVT. Despite not having an acute stroke unit in Hospital Y, stroke care pathway has been structured to allow patients to receive their bolus dose of IVT in the CT suite to shorten door-to-needle time. At the point of interviews, participants from Hospital Y and X attributed their challenges to create an acute stroke unit to having limited beds in their intensive care units.

*Lack of human resources*

Shortage of manpower, heavy workload and high turnover of staff were among the quoted barriers related to human resources which affected the provision of optimal care for IVT. As mentioned by one doctor (Z-09; IDI*): “Not enough manpower, very busy. You need to run a ward, you need to do rounds, you need to do discharge, you need to attend clinic and then suddenly (when) thrombolysis calls, you have to go and attend.”* Echoed by others: *“Workload issue. Sometimes when we are attending to patients, one needs to go for CT imaging, one comes in with stroke and the other has to have CPR. Which one do we attend to first?* (Y-03–01; FGD)*”*

Despite these challenges, higher authorities and the neurologists from Hospital Z took the effort to optimise available manpower and established a dedicated stroke team consisting of medical physicians, medical officers, and nurses, apart from the neurologists themselves to handle IVT. In Hospital Y, the absence of a stroke team has made stroke activations difficult at the point of the interview: *“It is quite (a) pity that they have the oncall person (neurologist or trainee) to see everyone. While they are halfway seeing one patient, suddenly there is an acute stroke and they must run there* (Y-07; IDI)*.”*

*Financial support for drug availability*

Limited budget to support drug availability for IVT were acknowledged. Furthermore, r-TPA has yet to be recognised as an emergency drug in the MOH, making it even more difficult to be readily available within ED. Hospital Z showed an example of how support from higher authorities and other departments have been crucial in maintaining the availability of drug (Z-01; IDI): *“every time we said we needed it, they (higher authorities) have never said “No”. Our usage exceeded many times off budget.”*

*Limited availability of data*

The availability of outcome-related IVT data remains limited despite many participants concurring that it is a useful evidence-based measure to prove the benefits of providing the service. The importance of having data to monitor the outcomes from IVT was mentioned: *“We update our hospital to tell them our outcomes. (From the data), we think we save the hospital from readmission, disability and then we shorten the hospital stay* (Z-01; IDI)*”* and *“I think data (basically) functions as a proof for people to see that this (IVT) works* (Y-02; IDI)*”*.

## Discussion

This comparative case study identified five factors that influenced the provision of IVT in the management of ischemic stroke across three public hospitals in Malaysia. Of those factors, our study highlights three factors which varied across these hospitals: leadership from the perspective of having quality stroke champions, team cohesiveness which entailed team dynamics and its degree of alignment, and facilitative work process which included workflow simplification and familiarity and experience on IVT. Two other factors which were consistently identified as barriers in these hospitals were patient factors which largely encompassed delayed presentation, and resource constraints. Supporting these results were review of the medical records which showed that more than half of our ischemic stroke patients missed the opportunity to receive IVT due to delayed presentation (50 – 67.6%).

Consistent with our findings, the pivotal role of leadership in strengthening the quality of healthcare has been proven, both in stroke care [12, 17] and in many health related-implementations [18]. Having good leadership in healthcare settings was associated with better team effectiveness in chronic illnesses and known to improve a multitude of health outcomes including guideline adherence and patient safety [18, 19]. Moreover, different types of leadership were observed to exert different influences on the provision of IVT. In our study, enthusiastic and motivated stroke champions have been observed to drive the service forward at a faster rate compared to leaders who tend to be more cautious about potential complications that may result from IVT. Similarly in Sweden, having authoritative leaders was regarded as an important element for successful implementation of IVT rather than those who prefer to get consensus for every matter [17].

Cohesiveness among team members was another factor that attributed to the variation in IVT rates. To augment team dynamics, engagements among team members via effective communication is crucial to promote interprofessional teamwork in managing integrated care pathways such as stroke care [12, 17]. Besides that, the option of responsibility sharing via training of non-neurologists to deliver IVT has been an attractive initiative especially in resource-poor countries where the proportion of neurologists to stroke patients is low [20]. Nevertheless, apart from heavier workload, different levels of receptiveness were present partly from the concern of possible complications or poorer patient outcomes among the non-neurologists despite reassuring evidences [21, 22].

The extent of how aligned team members were in respect to their perspectives on the risk and benefits of IVT have also contributed to team cohesiveness. Discordant beliefs and values is a common view shared in previous studies, where this is often attributable to the lack of knowledge and experience in handling IVT [17]. Consequently, physicians were reported to feel uncertain with outcomes from thrombolysis or to disapprove of the treatment option [7, 17].

Parallel to our findings on optimisation of workflow through simplification of work processes and providing aids, Huang et al. reported significant improvements of 1.4 to 2.3 times the odds of IVT uptake and reduction in time delays by having transportation and stroke protocols in place, establishing in-hospital organisational programmes as well as incorporating the use of technology [23]. Apart from that, having adequate familiarity and knowledge as well as a sense of urgency to handle IVT among healthcare providers were other essential elements to facilitate the work process. While familiarity and knowledge are commonly quoted to influence the implementation of IVT [12, 17, 24], less was known about the latter. In some studies where an inverse relationship between the onset-to-door and door-to-needle time was found, postulations of possible lack of urgency in patients who arrived earlier because their symptoms were milder or less critical compared to those who reached later were discussed [25]. Besides being associated to guideline awareness, cultivating the sense of urgency for IVT could depend on priority setting, both from the perspective of the individuals and the organisation.

Although the percentage of patients who arrived beyond 4.5 h of the therapeutic window time ranged widely from 31% in Greece [26] to 68% in Romania [27] and 75% in India [28], delayed presentation remained as the commonly cited patient-related barrier to the uptake of IVT worldwide, and consistently in our study. Multiple reasons were attributed to the delay, including lack of recognition of stroke symptoms, lack of awareness of the availability of IVT as a treatment option, and logistics challenges [29].

Similar to our findings, resource constraint is a commonly identified barrier to the implementation of IVT in other countries [6, 30]. Several challenges were reported, including limited physical space to establish stroke units, bed shortage, lack of imaging facilities, financial constraint and inadequate manpower. Although this issue spans worldwide, the extent of these challenges appeared to be greater in low- and middle-income countries [31]. Interestingly, our study shared how stroke care is optimised by innovative efforts to maximise the utilisation of existing resources, in coping with resource constraints. With support from their higher authorities, leaders from Hospital Z empowered and privileged other relevant healthcare providers to establish a dedicated stroke team and sectioned off a 6-cubicle area in their medical ward to form an acute stroke unit. One other exemplary case is in Thailand where a stroke fast track programme was established by similarly leveraging on non-neurologists to conduct IVT, creating a network referral system between stroke hospitals and hospitals that cannot provide IVT, and establishing telestroke consultation. This resulted in a 220% increase in terms of patient accessibility to the therapy between 2008 to 2013 [32].

The strength of our study lies in the methods. The choice of using a case study allowed an in-depth understanding of a multi-faceted complex issue in a real-life setting, which in the context of this study would be the factors influencing the provision of IVT in the management of ischemic stroke. Evidence from multiple case studies allows comparisons or replications to be made across cases and thus, is often regarded to be more robust [33]. The use of case study also allowed collection of data from multiple sources and triangulation of these information to strengthen the validity and reliability of our findings. To minimise interpretation bias, we conducted independent and cross coding processes by multiple coders before deriving the finalised themes. Regular meetings were held to discuss on coding similarities and differences. Additionally, the application of the TICD framework to guide the process of identifying the determinants of change in clinical practice further reinforced the credibility of our findings. Given that recruitment of the interview participants was conducted purposively, possibilities of selection bias may not be excluded but the bias is deemed minimal because findings comprised of a fair share of negative and positive experiences. Also, the percentages shown in Fig. 2 were based on information available from either medical or stroke registry records and thus, maybe affected by documentation bias.

### Implications to clinical practice, policy, and research

Findings are aimed to provide an insightful understanding in planning for targeted interventions to improve the provision of IVT in areas with similar settings. Previous recommendations which were adapted from the positive lessons learnt from our single case study were focused on strategies at respective organisation. This include adopting an interdisciplinary approach via behavioural interventions, optimising work processes for stroke service to identify areas that do not add value to patient outcomes, and providing opportunities for training and capacity building via innovative education [10].

Having understood the influencing factors and their relationship from a cross case comparison approach, potential strategies from the higher levels of the health systems should be deliberated. Great considerations should be placed into choosing the right leaders to manage stroke care. There are advantages and disadvantages of different leadership type and having a good mix of personalities would ensure optimal success in implementations [34]. One local intervention that runs parallel to this was a recently implemented initiative of electing state stroke champions in every state of Malaysia. This initiative is hoped to make stroke a priority nationwide and to decentralise the governance of stroke care services at state levels for decisions on stroke action plans to be tailored to the needs of the respective state [35]. To ensure its success, the choice of these stroke champions should ideally account for their leadership styles and capabilities, apart from their past commitments in stroke care.

From a community’s viewpoint, interventions targeted to improve patients’ delayed presentation should be given due consideration. Although the extent of its influence on the variation of IVT rate is potentially minimal from the consistent findings across three hospitals, the magnitude of its impact on overall IVT uptake calls for immediate actions to be put into place. Effects from public education or mass media campaigns to improve the emergency response for stroke however, remained varied thus far [36, 37]. In addition, specific groups may be more receptive to different interventions compared to the others; making it difficult and complex to develop an intervention that is suitable for the general population. One strategy however, that has been seen to deliver effective and sustainable impacts on recognition of stroke symptoms and their response was stroke education to school-going children [38–40]. Stroke education targeting children is anticipated to be effective and sustainable because inclusion of the programme into school curriculum would ensure that the information reach all ongoing school children in a simple manner. Nurturing their knowledge on stroke symptoms and response from young is likely to build strong awareness on stroke in the future [39]. In Malaysia, although the FAST Heroes education campaign to enhance community’s stroke preparedness through children’s education has recently been launched [41], more effort should be placed to ensure its continuous uptake and sustainability.

Eminently, implementation of these interventions should be planned synchronously with research to systematically evaluate their effectiveness and feasibility. Tying these interventions to an evidence-based component would allow monitoring of its sustainability and prevent wasteful healthcare spending.

## Conclusions

In addition to the global effort to explore sustainable measures to improve patients’ emergency response for stroke, attempts to improve the provision of IVT for stroke care should also consider the inclusion of interventions targeting on health systems perspectives such as promoting quality leadership, team cohesiveness and workflow optimisation.

### Supplementary Information

**Additional file 1. **Mapping of questions for interview guide to relevant Tailored Implementation for Chronic Disease (TICD) domains.

**Additional file 2. **Interview guide for neurologist/emergency physicians/medical officer.

## Data Availability

The dataset generated and analysed during the current study are not publicly available due to ethical and legal restrictions but are available from the corresponding author on reasonable request.

## Abbreviations

IVT: Intravenous thrombolysis
r-TPA: Recombinant tissue plasminogen activator
TICD: Tailored Implementation for Chronic Disease
MOH: Ministry of Health
IDI: In-depth interview
FGD: Focus group discussion
ED: Emergency department
HOD: Head of Department
