# Supplementary material for: Identifying factors in the provision of intravenous stroke thrombolysis in Malaysia: a multiple case study from the healthcare providers’ perspective
Source: BMC Health Serv Res. 2024 Jan 5;24:34. doi: 10.1186/s12913-023-10397-8 (PMC10768456; doi:10.1186/s12913-023-10397-8)
Supplement: Supplementary file 1 — Additional file 1. Mapping of questions for interview guide to relevant Tailored Implementation for Chronic Disease (TICD) domains. [file 12913_2023_10397_MOESM1_ESM.docx]

**Additional File 1.** Mapping of questions for interview guide to relevant Tailored Implementation for Chronic Disease (TICD) domains

| **Relevant TICD domains ^a, b^** | **Questions for interview guide** |
| --- | --- |
| Warmup / rapport building | Could you begin by telling me a little about your role, and how long you have worked, in the (xx) [ ] department |
|  | What do you do in this hospital in terms of stroke care? What is your experience with acute ischemic stroke patients?   - - What usually is being done for acute ischemic stroke cases presented to your hospital?   - Can you describe what happens during the process of administering intravenous thrombolytic therapy/thrombectomy in your hospital?   - How are stroke cases prioritised compared to other cases (in ED)? |
| Overall  Incentives and resources | What are the factors that would improve the uptake of thrombolytic therapy/thrombectomy in your hospital?   - - How do you think we can improve administration of acute reperfusion therapy to eligible stroke patients?   - What kind of resources would allow for increased use of thrombolytic therapy or thrombectomy? (personnel, access to CT, availability of drug)   - Do you have assistance needed to administer acute reperfusion therapy? (clinical supervision, teleconsultation, decision aids) |
| Overall  Social, political, and legal issues | What might be factors that hinder the use of acute reperfusion therapy in acute stroke?   - - Describe the barriers to administration of acute reperfusion therapies (within department/across departments)   - Other hospitals talk about budget being an issue, what is your opinion on this? |
| Professional interactions  Individual health professional factors | What is your opinion about the communication between team members within the department during management of acute stroke care?   - - How about communication between the departments?   - What is your opinion about sharing the responsibility to administer acute reperfusion therapy across departments and not solely by the neurologists?   - What changes (in terms of communication) do you think would benefit the implementation of acute reperfusion therapy in your hospital? |
| Guideline factors | Is there a standard protocol for administration of thrombolytic therapy or thrombectomy in stroke patients? What is your opinion on this?   - - How feasible is it to use stroke protocols in your practice?   - How are the stroke protocols consistent with the current workflow? |
| Guideline factors | How do you think having protocols have improved the administration of thrombolytic therapy and thrombectomy?   - - What would have made it more helpful? |
| Guideline factors | What is your opinion about the current guidelines for management of ischemic stroke in Malaysia?   - - How do you view the current guidelines?   - What would you say about how well you can rely on the information from the guidelines? |
| Individual health professional factors | What is your opinion about the knowledge and expertise needed to administer acute reperfusion therapy appropriately?   - - Do you think the colleagues in your department would have these mentioned skills? How about colleagues from other departments? |
| Individual health professional factors  Incentives and resources  Capacity of organizational change | What is your opinion about evidence that thrombolytic therapy/thrombectomy leads to better outcomes?   - What are your thoughts about the use of thrombolytic therapy or thrombectomy for patient outcomes? - We do not have much local data. How do you think the availability of local data would affect the uptake of acute reperfusion therapy? - What is your opinion about how much priority is being given to the administration of acute reperfusion therapy for ischemic stroke patients in comparison to other activities in the hospital? |
| Overall  Capacity for organizational change | Are there any other factors that you think might help or hinder the administration of thrombolytic therapy or thrombectomy in your hospital?   - - What do you think about the regulations and policies within your hospital? Are they supportive of the administration of acute reperfusion therapy in your hospital?   - What leadership or management support do you think is necessary to assist you and your colleagues in providing acute reperfusion therapy for stroke patients? |
| Capacity for organizational change | How much feedback do you receive about your management of hyperacute stroke patients?   - - How useful do you think monitoring and feedback/regular audits will be if it is available?   - What information would you be most interested to receive for your performance? |
| Patient factors | In your opinion, how do patients and family members perceive the administration of thrombolytic therapy/thrombectomy?   - - Do you think there is difficulties to get patients and family members to understand the benefits and risks from thrombolytic therapy or thrombectomy? |
| Wrap-up | What are the measures that have been put in place to improve the use of thrombolytic therapy in your hospital?   - - What are your experiences with those measures?   What recommendations would you like to make to the hospital authorities on how to improve hyperacute stroke care services in the hospital?   - - If there is one thing that you can change about the current system, what would it be and why?   - What do you value most in the current system?   Is there anything else I need to know to better understand the administration of acute reperfusion therapy and overall provision of hyperacute stroke care? |

*TICD: Tailored Implementation for Chronic Disease framework

References:

^a^ Skolarus et al 2019. Understanding determinants of acute stroke thrombolysis using the TICD framework: a qualitative study

^b^ Baatiema 2017 Barriers to evidence-based acute stroke care in Ghana_a qualitative study on the perspectives of stroke care professionals
