# Supplementary material for: Identifying factors in the provision of intravenous stroke thrombolysis in Malaysia: a multiple case study from the healthcare providers’ perspective
Source: BMC Health Serv Res. 2024 Jan 5;24:34. doi: 10.1186/s12913-023-10397-8 (PMC10768456; doi:10.1186/s12913-023-10397-8)
Supplement: Supplementary file 2 — Additional file 2. Interview guide for neurologist/emergency physicians/medical officer. [file 12913_2023_10397_MOESM2_ESM.docx]

**Additional File 2**. Interview guide for neurologist/emergency physician/medical officer.

***Introduction***

1. *Make sure all participants are available online and everyone can hear/speak*
2. *Introduce the research team*

*3. Obtain consent for participation and audio-taping the interview*

***Start audio-taping from here***

*4. Explain the objectives*

1. *To understand the experiences and challenges in the provision of hyperacute stroke care / to describe factors influencing provision of hyperacute stroke care*
2. *To evaluate the processes of care in the provision of hyperacute stroke care in Ministry of Health hospitals according to the clinical practice guidelines*

*5. Explain the process*

*a. The interview will take about 1 hour of the participant’s time*

*b. Interested to hear participant’s experience and challenges in delivering hyperacute stroke care services*

*c. No right or wrong answer; appreciate an honest opinion*

*d. Feel free to disagree/agree to any opinion proposed (FGD)*

*e. Mobile phones to be switched off during interview*

*f. Interview will be recorded for data analysis purposes but identity will be anonymised and confidentiality will be assured*

*g. Participants to mute themselves and only unmute upon answering questions (to reduce background noise for recording)*

*6. Any questions before we start?*

*7. Agreed to be interviewed and recorded for interview? (to get a verbal consent)*

**Question guide (Do not mention the probes until you are asked to clarify!)**

1. Could you begin by telling me a little about your role in [neurology/emergency] department
   - What is your role in this department? (general role)
   - How long have you worked in this department?
2. In recent time, what is your experience with an acute ischemic stroke patient?
   - If yes, describe - tell me about your experience
   - If no, reason – is there a specific reason
3. What usually is being done for acute ischemic stroke cases presented to your hospital? (If they do not start talking about thrombolysis, prompt them)
   - Tell me about the type of treatment available for acute ischemic stroke cases presented to your hospital?
   - Thrombolysis/thrombectomy
4. Can you describe what happens during the process of administering intravenous thrombolytic therapy/thrombectomy in your hospital?
   - Are there any workflow issues that you think affects the process?
   - Have you noticed anything that causes a delay in the process?
   - Have you noticed a difference in the process on weekdays and at weekends?
5. How are stroke cases prioritised compared to other neurology cases?
   - What kind of patients would you prioritize for immediate brain imaging/act fast in terms of management?
6. What are the factors that would improve the uptake of thrombolytic therapy/thrombectomy in your hospital?
   - How do you think we can improve administration of acute reperfusion therapy to eligible stroke patients?
   - What kind of resources would allow for increased use of thrombolytic therapy or thrombectomy? *(personnel, access to CT, availability of drug)*
   - Do you have assistance needed to administer acute reperfusion therapy? *(clinical supervision, teleconsultation, decision aids)*
7. What might be factors that hinder the use of acute reperfusion therapy in acute stroke?
   - Describe the barriers to administration of acute reperfusion therapies (within department/across departments)
   - Other hospitals talk about budget being an issue, what is your opinion on this?
8. What is your opinion about the communication between team members within the department during management of acute stroke care?
   - How about communication between the departments?
   - What is your opinion about sharing the responsibility to administer acute reperfusion therapy across departments and not solely by the neurologists?
   - What changes (in terms of communication) do you think would benefit the implementation of acute reperfusion therapy in your hospital?
9. Is there a standard protocol for administration of thrombolytic therapy or thrombectomy in stroke patients? What is your opinion on this?
   - How feasible is it to use stroke protocols in your practice?
   - How are the stroke protocols consistent with the current workflow?
10. How do you think having protocols have improved the administration of thrombolytic therapy and thrombectomy?
    - What would have made it more helpful?
11. Are there any other factors that you think might help or hinder the administration of thrombolytic therapy or thrombectomy in your hospital?
    - What do you think about the regulations and policies within your hospital? Are they supportive of the administration of acute reperfusion therapy in your hospital?
    - What leadership or management support do you think is necessary to assist you and your colleagues in providing acute reperfusion therapy for stroke patients?
12. What is your opinion about the knowledge and expertise needed to administer acute reperfusion therapy appropriately?
    - Do you think the colleagues in your department would have these mentioned skills? How about colleagues from other departments?
13. What is your opinion about evidence that thrombolytic therapy/thrombectomy leads to better outcomes?

- What are your thoughts about the use of thrombolytic therapy or thrombectomy for patient outcomes?
- We do not have much local data. How do you think the availability of local data would affect the uptake of acute reperfusion therapy?
- What is your opinion about how much priority is being given to the administration of acute reperfusion therapy for ischemic stroke patients in comparison to other activities in the hospital?

1. What is your opinion about the current guidelines for management of ischemic stroke in Malaysia?
   - How do you view the current guidelines?
   - What would you say about how well you can rely on the information from the guidelines?
2. How much feedback do you receive about your management of hyperacute stroke patients?
   - How useful do you think monitoring and feedback/regular audits will be if it is available?
   - What information would you be most interested to receive for your performance?
3. In your opinion, how do patients and family members perceive the administration of thrombolytic therapy/thrombectomy?
   - Do you think there is difficulties to get patients and family members to understand the benefits and risks from thrombolytic therapy or thrombectomy?
4. What are the measures that have been put in place to improve the use of thrombolytic therapy in your hospital?
   - What are your experiences with those measures?
5. What recommendations would you like to make to the hospital authorities on how to improve hyperacute stroke care services in the hospital?
   - If there is one thing that you can change about the current system, what would it be and why?
   - What do you value most in the current system?
6. Is there anything else I need to know to better understand the administration of acute reperfusion therapy and overall provision of hyperacute stroke care?

Thank you so much for taking the time to talk with us today. If you have any questions about what we discussed today, or would like to add or change anything at a later time, feel free to contact us.
